# V-ATPase A Is a Key Protein Involved in the Toxicity of *Bacillus thuringiensis* Cry39Ab1 in *Bradysia odoriphaga* (Diptera: Sciaridae)

**DOI:** 10.3390/insects17060563

**Published:** 2026-05-29

**Authors:** Shuo Feng, Yizhuo Zhang, Jiaxu Cheng, Weiping Cao, Shengqiang Shen, Qingjun Wu, Jun Cai, Jian Song

**Affiliations:** 1 Plant Protection Institute, Hebei Academy of Agriculture and Forestry Sciences, Baoding 071000, China; fengshuo9311@163.com (S.F.); chengjx8023@163.com (J.C.); cwplx751209@163.com (W.C.); shenshengqiang@pku.edu.cn (S.S.); 2 National Collection of Plant-Associated Microbes (Hebei), Baoding 071000, China; 3 IPM Innovation Center of Hebei Province, Baoding 071000, China; 4 International Science and Technology Joint Research Center on IPM of Hebei Province, Baoding 071000, China; 5 Department of Microbiology, College of Life Sciences, Nankai University, Tianjin 300071, China; 1120230727@mail.nankai.edu.cn; 6 State Key Laboratory of Vegetable Biobreeding, Institute of Vegetables and Flowers, Chinese Academy of Agricultural Sciences, Beijing 100081, China; wuqingjun@caas.cn

**Keywords:** V-ATPase A, Cry39Ab1, *Bradysia odoriphaga*, *Bacillus thuringiensis*, toxicity

## Abstract

*Bradysia odoriphaga* is a devastating soil pest of *Allium tuberosum* (Chinese chive), and current control relies heavily on chemical insecticides. Cry39Ab1 toxins from *Bacillus thuringiensis* (Bt), which are highly toxic to *B. odoriphaga*, offer an environmentally friendly alternative. However, its mechanism of action remains unclear. In this study, we identified the involvement of vacuolar H^+^-ATPase subunit A (V-ATPase A) in Cry39Ab1 insecticidal activities. The full-length cDNA sequences of *BoV-ATPase A* was contained 1659 bp open reading frame (ORF), encoding a protein of 552 amino acids with a calculated molecular weight of 59 kDa and an isoelectric point of 9.11. Successful expression and purification of BoV-ATPase A (with GST and His tags) and Cry39Ab1 (with GST and His tags) proteins were achieved. GST pull-down assays demonstrated a direct interaction between recombinant BoV-ATPase A and activated Cry39Ab1 toxin *in vitro*. Heterologous expression of BoV-ATPase A in Cry-insensitive Sf9 cells conferred susceptibility to Cry39Ab1, resulting in a significant increase in cytolysis compared with control cells. Finally, RNAi-mediated knockdown of *BoV-ATPase A* in larvae significantly decreased their susceptibility to Cry39Ab1, as evidenced by a marked decrease in mortality. This is the first report that BoV-ATPase A is a key protein required for Cry39Ab1 toxicity, revealing its insecticidal mechanism and establishing BoV-ATPase A as a potential target for pest control.

## Simple Summary

*Bradysia odoriphaga* is an important underground pest, currently controlled mainly by chemical insecticides. The natural insecticidal protein Cry39Ab1 from *Bacillus thuringiensis* (Bt) offers a friendly alternative, but its mode of action is unclear. Here, the results showed that GST pull-down assays demonstrated a direct interaction *in vitro*. Expressing BoV-ATPase A in Cry-insensitive Sf9 cells made them susceptible to Cry39Ab1. RNAi knockdown of *BoV-ATPase A* in larvae significantly decreased their susceptibility to Cry39Ab1. This is the first report that BoV-ATPase A is a key protein required for Cry39Ab1 toxicity, revealing its insecticidal mechanism and establishing BoV-ATPase A as a potential target for pest control.

## 1. Introduction

*Bradysia odoriphaga* (Diptera: Sciaridae) is a devastating soil-dwelling pest of Chinese chive, causing substantial economic losses in China [1,2]. Its current control relies heavily on chemical insecticides, which inevitably leads to serious consequences, including the evolution of *B. odoriphaga* resistance to insecticides, environmental pollution, and food safety risks [3,4,5]. These challenges underscore an urgent need to develop sustainable and friendly alternative control strategies.

*Bacillus thuringiensis* (Bt) produces crystal proteins (Cry) that exhibit specific insecticidal activity against lepidopteran, dipteran, and coleopteran pests, making it a cornerstone of global agricultural pest biocontrol and driving the widespread application of microbial insecticides and transgenic crops [6,7,8]. The insecticidal mechanism of Cry proteins is primarily interpreted through two prevailing models: the former centers on physical disruption of the midgut membrane, resulting in osmotic imbalance and cell lysis, whereas the latter involves receptor-mediated activation of intracellular signaling cascades that ultimately lead to programmed cell death. Despite their divergent pathways, both models share a fundamental prerequisite: the specific binding of Cry toxins to receptors on the insect midgut epithelium [9,10,11]. Currently identified Cry protein receptors mainly include alkaline phosphatase (ALP), cadherin (CAD), aminopeptidase N (APN), and ATP-binding cassette (ABC) transporters [9,12]. The vacuolar H^+^-ATPase (V-ATPase) is a multi-subunit protein complex comprising a cytoplasmic V1 sector and a membrane-embedded V_0_ [13]. It functions primarily as an ATP-driven proton pump and is critically involved in diverse cellular processes, including endocytosis, lysosomal degradation, secondary transport, TOR signaling, ion translocation, and energy metabolism [14]. The soluble V_1_ consists of eight subunits (A–H), whereas the V_0_ is composed of subunits a, c8, c′, c″, d and e. Structural and biochemical studies suggest that the V_1_ may interact directly with Cry proteins, pointing to a potential functional interface [15,16]. However, the potential role of V-ATPase, a class of functionally crucial membrane proteins widely expressed in the insect midgut, in the recognition of Cry proteins and the transduction of their toxicity remains insufficiently elucidated. The related molecular mechanisms and direct functional evidence are still largely lacking.

Emerging evidence supports the role of specific V-ATPase subunits in the virulence process of Cry toxins. In *Anthonomus grandis*, V-ATPase A is identified as a putative binding partner for Cry8Ka5 [17], and similarly in *Aedes aegypti* for Cry11Aa [18]. In *Helicoverpa armigera*, V-ATPase B has been shown to interact with Cry1Ac, with its expression significantly down regulated in Cry1Ac resistant strains [19]. In contrast, subunit E specifically binds activated Cry2Ab toxin as confirmed by ligand blotting and competitive binding assays, a functional role further supported by cytotoxicity and bioassay data [20]. V-ATPase A contributes to the action of Cry1Ab/1Ac, Cry2Aa and Cry1Ca toxins in *Chilo suppressalis* [21]. Functional studies further demonstrated that RNAi silencing of the *V-ATPase B* in *Spodoptera exigua* reduced larval susceptibility to Cry2Aa [22]. Knockdown of the *V-ATPase G* gene in *Plutella xylostella* increased larval susceptibility to Cry1Ac, indicating a complex specific modulation of the toxin response [23].

While the insecticidal activity of Cry39Aa1 against *Anopheles stephensi* and its binding to midgut brush border membrane vesicles (BBMV) were initially reported by Ito [24], its specific receptor remained unknown. Cry39Ab1, a homolog sharing 94.10% amino acid identity with Cry39Aa1, has recently been shown to be active against *B. odoriphaga* [25]. However, its molecular mode of action remains unclear. Through *in vitro* binding assays, cytotoxicity tests in Sf9 cells, and *in vivo* RNA interference experiments, we provide direct evidence that BoV-ATPase A is essential for Cry39Ab1 toxicity.

## 2. Materials and Methods

### 2.1. Insect Rearing and Cell Line

The larvae of *B. odoriphaga* were collected from the Chinese chive fields of Baoding City, Hebei Province. The larvae were reared on an artificial diet under conditions of 25 ± 1 °C and 75% relative humidity, and healthy individuals of the same age were selected as the test insects.

*Spodoptera frugiperda* oocytes (Sf9) cells were cultured at 28 °C in Sf-900^TM^II SFM (all from Thermo Fisher, Shanghai, China) culture medium, supplemented with 10% heating activated bovine serum, 50 μg/mL streptomycin, and 50 U/mL penicillin (all from Solarbio, Beijing, China).

### 2.2. RNA Preparation and cDNA Synthesis

Total RNA from all samples was extracted using TRIzol (Takara Biomedical Technology, Beijing, China). cDNA templates were synthesized using the HiScript^®^III RT SuperMix for qPCR (+gDNA wiper) (Takara Biomedical Technology, Beijing, China) according to the manufacturer’s instructions. The synthesized cDNA was immediately stored at 20 °C for later use.

### 2.3. BoV-ATPase A Gene Cloning and Analysis

The gene encoding *BoV-ATPase A* was amplified using the primers BoV-ATPase A-F and BoV-ATPase A-R (Table 1) from the larva cDNA samples. The PCR product was purified and ligated into the pMD18-T vector (Takara, Kyoto, Japan), and the resulting construct was transformed into *E. coli* DH5α cells (TransGen Biotech, Beijing, China). Positive clones were selected via blue-white screening using X-gal and IPTG. White colonies were cultured, and colony PCR was performed using M13 primers (Table 1) with bacterial liquid as the template. Clones yielding PCR products of the expected size were sent to Tsingke Biotechnology Co., Ltd. (Tianjin, China) for sequencing.

All primers used in this study were designed using the NCBI Primer-BLAST tool (https://www.ncbi.nlm.nih.gov/tools/primer-blast/) (accessed on 20 September 2024) and subsequently synthesized by Tsingke Biotechnology Co., Ltd. (Tianjin, China). The amino acid sequence of the *BoV-ATPase A* gene was predicted online using the ExPASy Translate tool (https://web.expasy.org/translate/) (accessed on 30 September 2024), and its protein domains were analyzed via SMART (https://smart.embl.de/). To evaluate the homology of BoV-ATPase A with orthologs from other species, amino acid sequences of BoV-ATPase A from various insects were retrieved from the NCBI database. A phylogenetic tree was constructed using the neighbor-joining method in MEGA 11 software.

### 2.4. BoV-ATPase A and Cry39Ab1 Protein Expression and Purification

Cry39Ab1 was a novel insecticidal protein with insecticidal activity against *B. odoriphaga* discovered by our laboratory. After being submitted to BPPRC (https://www.bpprc-db.org/), it was named Cry39Ab1 (GenBank: PV231409).

To construct recombinant plasmids encoding BoV-ATPase A and Cry39Ab1 fused with glutathione S-transferase (GST) and polyhistidine (His) tags, two pairs of gene-specific primers were designed for each target: gst-V-ATPase A-F/R and his-V-ATPase A-F/R for BoV-ATPase A, and gst-cry39Ab1-F/R and his-cry39Ab1-F/R for Cry39Ab1 (Table 1). The purified PCR products were separately ligated into the pGEX-4T-1 (for GST fusion) and pET-28a (for His fusion) vectors using a seamless cloning kit (all from Takara, Kyoto, Japan). The ligation products were transformed into *E. coli* BL21(DE3) competent cells (Tiangen Biotech, Beijing, China) and plated on LB agar supplemented with either ampicillin (100 µg/mL) or kanamycin (50 µg/mL) (all from Solarbio, Beijing, China), then incubated overnight at 37 °C. Single colonies were picked and screened by colony PCR with vector-specific primers (pGEX-5, pGEX-3, T7, and T7-ter; Table 1). Clones yielding PCR products of the expected size were cultured on a larger scale for plasmid extraction, and the purified plasmids were verified by Sanger sequencing (Tsingke Biotechnology Co., Ltd., Tianjin, China).

The recombinant strains were cultured overnight at 37 °C. A 1% (*v*/*v*) inoculum was transferred into 200 mL of LB medium containing the corresponding antibiotic and incubated with shaking at 37 °C until the OD_600_ reached 0.6–0.8. Protein expression was induced by adding IPTG to a final concentration of 0.5 mmol·L^−1^, followed by incubation at 16 °C overnight with shaking. The bacterial cells were harvested by centrifugation at 6000 rpm for 10 min and resuspended in 15 mL of either PBS (for GST-tagged proteins) or lysis buffer (for His-tagged proteins). Cell disruption was performed by sonication using a 3 s pulse/8 s pause cycle, repeated 80 times. After sonication, the lysate was centrifuged at 12,000 rpm for 30 min at 4 °C to collect the supernatant.

The supernatant was loaded onto a column pre-packed with either GST resin or Ni-NTA resin. The flow-through fraction was collected, after which the column was washed with washing buffer (PBS for GST tags; lysis buffer containing 0.068 g imidazole per 100 mL for His tags). Bound proteins were eluted with elution buffer (50 mL ultrapure water containing 0.3 g Tris and 0.6 g reduced glutathione for GST tags; 100 mL lysis buffer containing 1.7 g imidazole for His tags). The eluted protein fractions were dialyzed against a buffer composed of 20 mM Tris-HCl (pH 7.5), 150 mM NaCl, and 1 mM DTT (all from Sigma-Aldrich, St. Louis, MO, USA). Protein concentration was determined using the BCA assay (Beyotime Biotechnology, Shanghai, China). The purified proteins were mixed with 5× SDS loading buffer, boiled for 7 min, and centrifuged at 12,000 rpm for 5 min before being analyzed by SDS-PAGE.

### 2.5. GST Pull-Down Validation and Western Blot

The experiment was conducted in two groups: (1) using GST-Cry39Ab1 as bait and HIS-BoV-ATPase A as prey; and (2) using GST-BoV-ATPase A as bait and His-Cry39Ab1 as prey. For each group, a negative control was set up using the corresponding GST tag protein alone. The procedure was as follows: 40 µL of glutathione agarose beads (Smart-Lifesciences, Changzhou, China), pre-equilibrated with binding buffer (20 mM Tris-HCl, pH 7.5, 150 mM NaCl, 0.5% NP-40, 1 mM DTT), were incubated with 2 µg of purified GST-Cry39Ab1 or GST-BoV-ATPase A protein (experimental groups), or an equimolar amount of GST protein (control groups), at 4 °C for 2 h with gentle rotation to immobilize the bait proteins on the beads. Subsequently, the beads were washed three times with binding buffer to remove non-specific binding. An equivalent amount (2 µg) of the corresponding prey protein (His-BoV-ATPase A or His-Cry39Ab1) was then added to each sample, followed by further incubation at 4 °C for 2 h. After incubation, the beads were washed five times with binding buffer. Finally, the bound complexes were eluted by boiling in 2× SDS-PAGE loading buffer for 5 min.

The treated samples were separated by 12% SDS-PAGE electrophoresis. Proteins were then transferred onto a polyvinylidene difluoride (PVDF) membrane (Millipore, Burlington, MA, USA) using the wet transfer method under the following conditions: constant current of 300 mA for 90 min in an ice-cooled transfer buffer (25 mM Tris, 192 mM glycine, 20% methanol). After transfer, the membrane was blocked with 5% non-fat milk (Beyotime Biotechnology, Shanghai, China) in TBST buffer (20 mM Tris-HCl, pH 7.6, 150 mM NaCl, 0.1% Tween-20) at room temperature for 1 h to prevent nonspecific binding. The membrane was then washed three times quickly with TBST buffer. Subsequently, it was incubated with a mouse anti-His tag or rabbit anti-GST tag monoclonal antibody (Abcam, Cambridge, UK) (diluted 1:3000) at 4 °C for 1.5 h. After primary antibody incubation, the membrane was washed three times with TBST buffer for 10 min each at room temperature to remove unbound primary antibodies. The membrane was then incubated with a secondary antibody (goat anti-mouse or goat anti-rabbit, diluted 1:5000) at room temperature for 1 h, followed by three 10 min washes with TBST buffer. Finally, the membrane was evenly covered with enhanced chemiluminescence (ECL) substrate (Thermo Fisher Scientific, Waltham, MA, USA) and exposed for image acquisition using an Image Quant LAS4000mini system (GE Healthcare, Tokyo, Japan).

### 2.6. RT-PCR Analysis

To investigate the expression profile of the *BoV-ATPase A* gene across different developmental stages of *B. odoriphaga*, samples of 1–4 st larvae and pupae were collected. Three biological replicates were prepared for each developmental stage. Total RNA was extracted from all samples, and 1 μg of RNA from each replicate was reverse-transcribed into cDNA for subsequent quantitative real-time PCR (qRT-PCR) analysis. *RPL18* and *EF-1α* of *B. odoriphaga* were selected as reference genes for normalization (Table 1).

The cDNA of all samples was analyzed by qRT-PCR in a 20 μL reaction system. Includes 2× ChamQ Universal SYBR qPCR Master Mix (SYBR Green) (Tiangen, Beijing, China) 10 μL, 0.4 μL forward primer and 0.4 μL reverse primer (10 μM), 1 μL cDNA, and 8.2 μL ddH_2_O using an Applied Biosystems 7500 Fast Real-Time PCR system (ABI, Carlsbad, CA, USA). Thermocycler conditions were 95 °C for 60 s, followed by 40 cycles of 95 °C for 5 s and 60 °C for 15 s. Triplicates were performed for each PCR reaction. 2^−ΔΔCT^ methods were used to calculate the relative expression [26].

### 2.7. Double-Stranded (dsRNA) Synthesis and RNAi

*BoV-ATPase A* dsRNA was synthesized using the AmpliScribe^TM^ T7-Flash^TM^ Transcription Kit (Lucigen Simplifying Genomics, Middleton, WI, USA). Specific dsRNA primers were listed in Table 1. PCR was performed with the procedures of 94 °C for 5 min, followed by 35 cycles of 94 °C for 30 s, 57 °C for 30 s, and 72 °C for 30 s, with a final extension at 72 °C for 10 min. Ethanol precipitation and nuclease-free water were utilized to purify and elute dsRNA. Double strands RNA of green fluorescent protein (dsGFP) was used as the non-target negative control.

RNAi was conducted according to the protocol described by Chen et al. [27]. Second-instar larvae were fed an artificial diet supplemented with dsBoV-ATPase A or dsGFP at a concentration of 30 µg/g diet. To assess RNAi efficacy, larvae were sampled at 24, 48, and 72 h post-treatment, and transcript levels of the target gene were quantified by qRT-PCR (ABI, Carlsbad, CA, USA). Each treatment consisted of at least 3 biological replicates, each containing 20 second-instar larvae.

### 2.8. Cry39Ab1 Protein Insecticidal Activity

To determine whether RNAi-mediated silencing of the *BoV-ATPase A* affects the insecticidal activity of Cry39Ab1, six experimental treatments were established. Each treatment consisted of at least 4 biological replicates, each containing 20 second-instar larvae. The treatments were designed as follows: (1) normal artificial diet; (2) dsGFP + normal artificial diet; (3) dsBoV-ATPase A + normal artificial diet; (4) artificial diet supplemented with Cry39Ab1; (5) dsGFP + artificial diet supplemented with Cry39Ab1; (6) dsBoV-ATPase A + artificial diet supplemented with Cry39Ab1. The Cry39Ab1 protein was used at a final concentration of 200 µg/mL in the artificial diet for bioassay. Larvae in all groups were maintained under controlled conditions (25 ± 1 °C, 75% RH). Mortality and phenotypic responses were recorded at 24 h intervals until 96 h post-treatment. Samples for RNA extraction and qRT-PCR validation of *BoV-ATPase A* gene knockdown were collected at 48 h after the onset of dsRNA feeding.

### 2.9. Cell Transfection

Sf9 cells were cultured in Sf-900™ II SFM medium within a humidified incubator maintained at 28 °C with ambient air exchange. For transfection, cells were seeded onto coverslips in 12-well plates at a density of 8 × 10^4^ cells per well. On the following day, plasmid constructs—either the empty vector (pFastBac1-Flag) or the overexpression vector (pFastBac1-V-ATPase A-Flag)—were diluted in jetPRIME buffer (Polyplus-transfection, Illkirch, France), vortexed for 10 s, and incubated for 5 min. Subsequently, jetPRIME reagent was added, followed by brief vortexing (5 s) and incubation at 28 °C for 10 min. The transfection complexes were then gently combined with OPTI-MEM medium (Gibco, Grand Island, NY, USA) and applied dropwise to the cell culture. After 6 h of incubation, the transfection mixture was replaced with fresh Sf-900™ II SFM medium. To confirm transfection efficiency, a subset of cells was examined under a fluorescence microscope (Leica DMi8, Wetzlar, Germany) post-transfection, wherein successful transfection was indicated by the presence of a green fluorescent signal.

Sf9 cells transfected with either the empty vector (pFastBac1-Flag) or the overexpression vector (pFastBac1-V-ATPase A-Flag) were harvested and washed once with PBS. Following washing, the cell pellet was collected by centrifugation. The cells were subsequently lysed in freshly prepared RIPA buffer (Beyotime, Shanghai, China) at 4 °C for 30 min. The resulting lysates were then centrifuged at 12,000× *g* for 15 min at 4 °C to remove cellular debris. Finally, the clarified supernatants were collected for subsequent Western blot analysis to assess protein expression levels.

### 2.10. Cytotoxicity Assay

Cytotoxicity assays were performed to evaluate the sensitivity of Sf9 cells expressing the V-BoV-ATPase A protein to Cry39Ab1 exposure. Sf9 cells stably transfected with BoV-ATPase A were incubated with activated Cry39Ab1 protein at final concentrations of 10 μg·mL^−1^ and 100 μg·mL^−1^ for 3 h at 28 °C. Following treatment, cell viability was assessed by staining with 0.4% trypan blue (Beyotime, Shanghai, China) and subsequently counting dead cells (identified by blue staining) under an optical microscope (Olympus, Tokyo, Japan) at 400× magnification. Sf9 cells transfected with the empty vector (pFastBac1-Flag) served as the negative control. The number of dead (blue-stained) and viable (unstained) cells in each well was quantified under a light microscope (Leica, Wetzlar, Germany) to calculate the cell mortality rate. Each transfection condition was independently assayed across 4–5 biological replicates.

### 2.11. Statistical Analysis

Data were expressed as mean ± SEM. All analyses were performed using SPSS 27.0 (IBM, Armonk, NY, USA). Normality and homogeneity of variance were tested using Shapiro–Wilk and Levene’s tests, respectively. Differences among multiple groups were assessed by one-way ANOVA, followed by Tukey’s HSD test for all pairwise comparisons or Dunnett’s test for comparisons with a single control group. Cell mortality in the cytotoxicity assay was calculated as described by Wei et al. (2019) [28]. A *p*-value < 0.05 was considered statistically significant.

## 3. Results

### 3.1. Cloning and Phylogenetic Analysis

**Figure 1:**
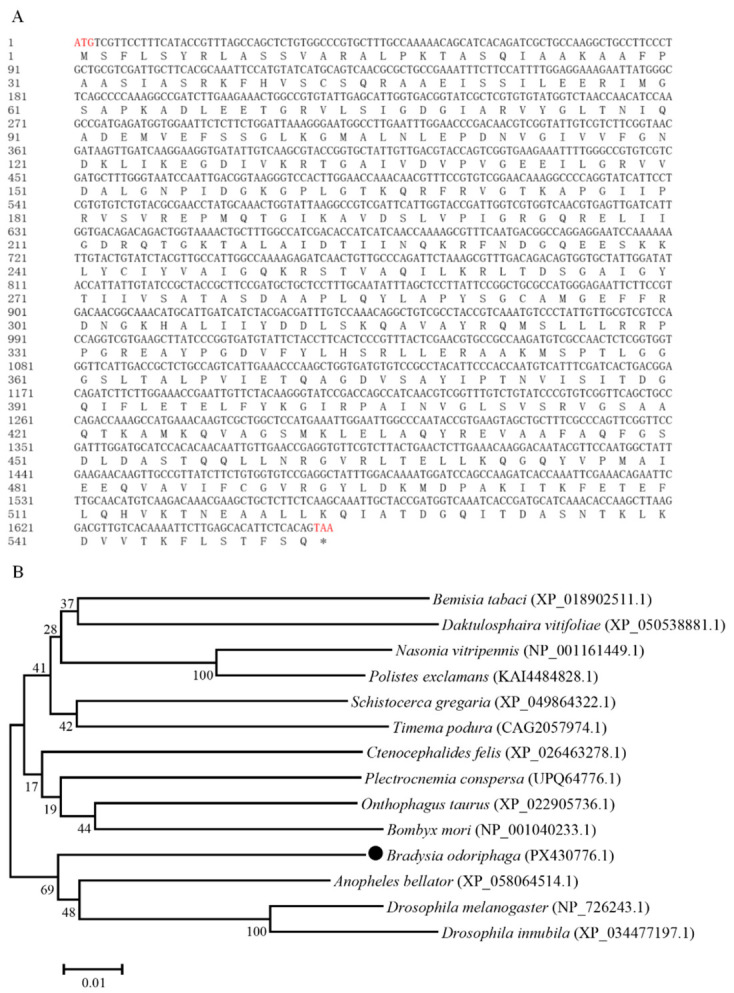
Complete cDNA sequences of *B. odoriphaga BoV-ATPase A* and a neighbor-joining phylogenetic tree generated by MEGA 7.0 of BoV-ATPase A amino acid sequences from different insect species. (**A**) Red font of “ATG” denote initiation codon. The asterisk and red font of “TAA” denote termination codon. (**B**) Bootstrap values, expressed as percentages of 1000 replications, are shown at branch points.

The cDNA of *BoV-ATPase A* (GenBank: PX430776) is 1659 bp in length and encodes a protein of 552 amino acids (Figure 1A). The predicted molecular weight and theoretical isoelectric point of the protein are approximately 59 kDa and 9.11, respectively. A phylogenetic tree was constructed using MEGA 11 based on the alignment of V-ATPase A amino acid sequences from dipteran, lepidopteran, and other insect species (Figure 1B). The phylogenetic analysis revealed that BoV-ATPase A is most closely related to *Anopheles bellator* (XP_058064514.1), *Drosophila melanogaster* (NP_726243.1), and *Drosophila innubila* (XP_034477197.1), indicating its evolutionary conservation among these dipteran insects.

### 3.2. BoV-ATPase A Expression in B. odoriphaga

**Figure 2:**
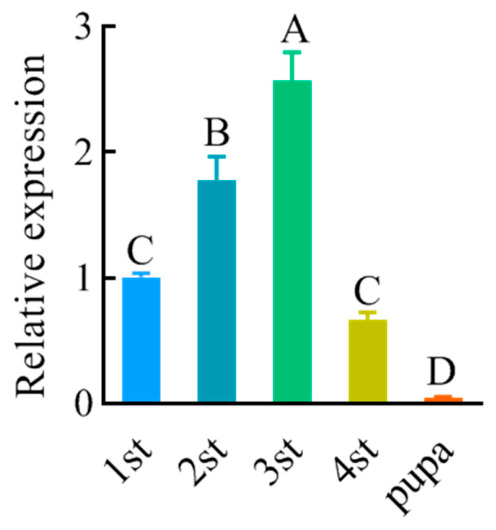
Expression of *BoV-ATPase A* gene at different developmental stages. Bars with different capital (*p* < 0.05) letters differ significantly.

The expression pattern of *BoV-ATPase A* was investigated across various developmental stages of *B. odoriphaga*, including 1st to 4th-instar larvae and pupae. qRT-PCR analysis revealed that the transcriptional level of *BoV-ATPase A* peaked during the third-instar larval stage, showing a significant difference compared with other stages (*p* < 0.05). This was followed by the second-instar larvae, and then the first-instar larvae, the latter of which exhibited no significant difference in expression compared with the fourth-instar larvae (*p* > 0.05). The lowest expression was observed in the pupal stage (Figure 2).

### 3.3. Prokaryotic Expression and Pull-Down Analysis of BoV-ATPase A and Cry39Ab1

**Figure 3:**
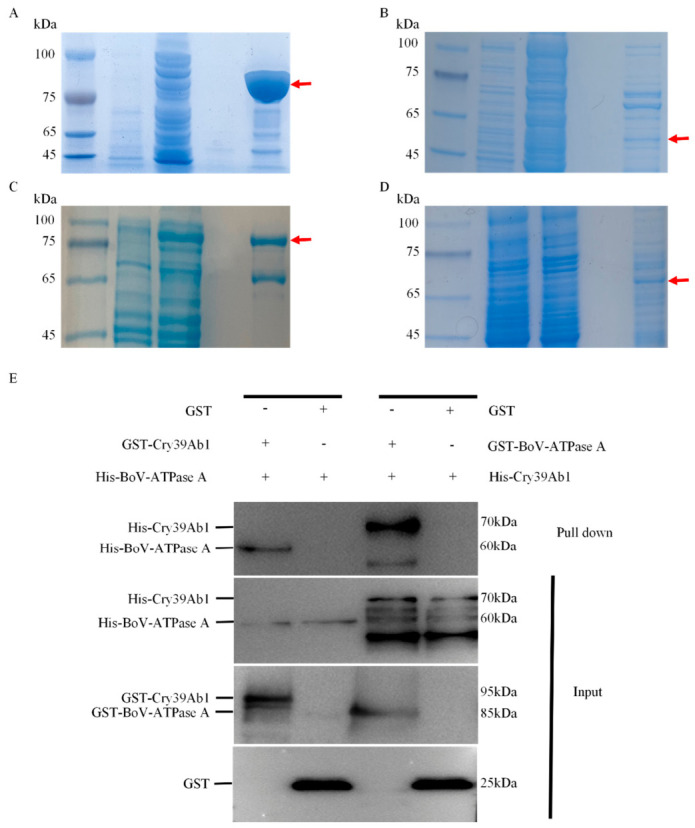
Prokaryotic expression and pull-down analysis of BoV-ATPase A and Cry39Ab1. (**A**) Expression and purification of the GST-BoV-ATPase A fusion protein. (**B**) Expression and purification of the His-BoV-ATPase A fusion protein. (**C**) Expression and purification of the GST-Cry39Ab1 fusion protein. (**D**) Expression and purification of the His-Cry39Ab1 fusion protein. From left to right: M: Marker; Lane 1: whole cell lysate without IPTG induction; Lane 2: Flow-through fraction; Lane 3: Wash fraction; Lane 4: purified protein. The red arrows indicate the target protein after purification. (**E**) GST pull-down assay validating the interaction between BoV-ATPase A and Cry39Ab1.

The prokaryotic expression of BoV-ATPase A was carried out using the pGEX-4T-1 (GST-tag) and pET-28a (His-tag) vectors. Recombinant plasmids pGEX-BoV-ATPase A and pET28a-BoV-ATPase A were successfully constructed via seamless cloning. Following induction with IPTG, SDS-PAGE analysis confirmed the successful expression of BoV-ATPase A in *E. coli* BL21(DE3) cells. The apparent molecular weights of the fusion proteins were approximately 85 kDa (Figure 3A, GST-tagged) and 60 kDa (Figure 3B, His-tagged), consistent with their theoretical predictions.

To investigate whether a direct interaction exists between Cry39Ab1 and BoV-ATPase A, we constructed the recombinant plasmids pGEX-Cry39Ab1 and pET28a-Cry39Ab1. SDS-PAGE analysis confirmed the successful expression of Cry39Ab1 in BL21(DE3) cells following IPTG induction, with apparent molecular weights of approximately 95 kDa (Figure 3C) and 70 kDa (Figure 3D).

A GST Pull-down assay was subsequently performed. GST-fusion proteins immobilized on glutathione-sepharose beads were used as bait and incubated with the corresponding His-tagged proteins in solution. Western blot analysis (Figure 3E, File S1) revealed specific binding signals in both reciprocal pull-down experiments: when GST-Cry39Ab1 was used as bait, it effectively pulled down His-BoV-ATPase A, and a clear His-specific band was detected (lane 2). Conversely, when GST-BoV-ATPase A served as bait, it specifically pulled down His-Cry39Ab1 (lane 4). In negative control experiments, GST alone showed no binding to either His-BoV-ATPase A or His-Cry39Ab1, and no His signal was detected in the corresponding lanes (lanes 1 and 3). These consistent and mutually reinforcing results from reciprocal experiments provide strong evidence that Cry39Ab1 and BoV-ATPase A can directly and specifically interact *in vitro*.

### 3.4. Overexpression of BoV-ATPase A Enhances the Susceptibility of Sf9 Cells to Cry39Ab1

**Figure 4:**
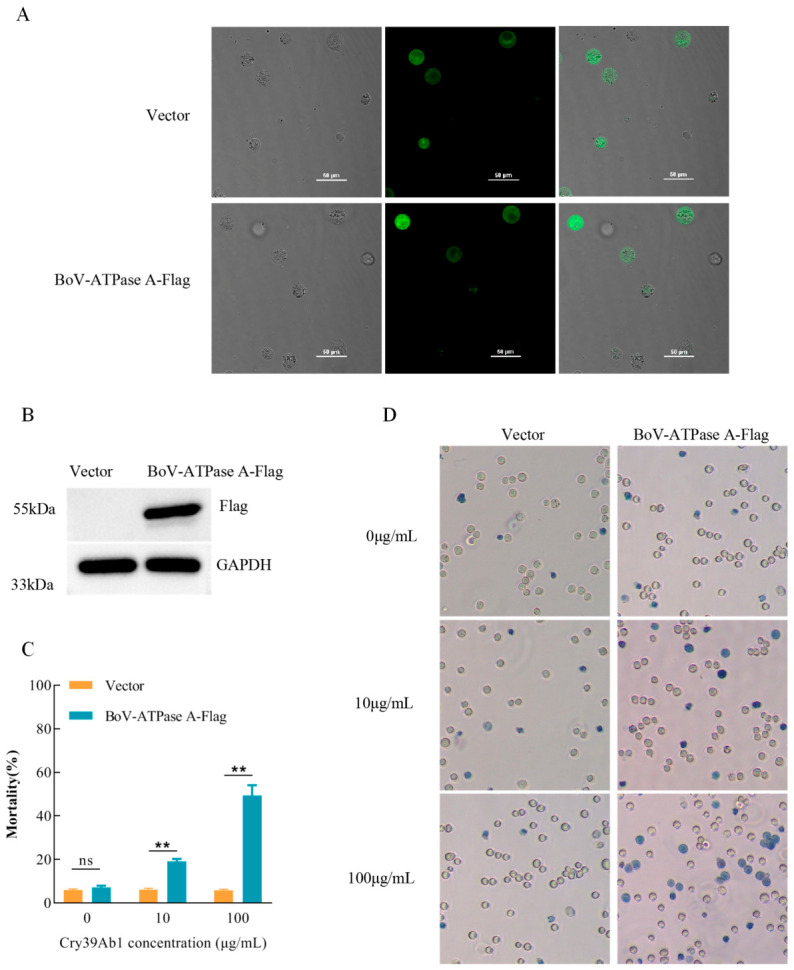
Cytotoxicity of Cry39Ab1 toxin protein at different concentrations to *BoV-ATPase A* Sf9 cells. (**A**) Transfected pFastBac1-Flag and pFastBac1-BoV-ATPase A-Flag in Sf9 cells. (**B**) Analysis of BoV-ATPase A expression in the transfected cells by Western blot. (**C**) Change in BoV-ATPase A-transfected Sf9 cell mortality after Cry39Ab1 toxin protein treatment. (**D**) Sensitivity of Sf9 expressing BoV-ATPase A protein to Cry39Ab1 toxin protein. The photographs were taken at 100× under an inverted microscope. The dead cells were stained blue with trypan blue. The “ns” indicates no significant difference, and ”**” shows significant differences in cell mortalities between each treatment (*p* < 0.01, Student’s *t*-test, DPSSOFT: DPS7.05).

Western blot and fluorescence imaging confirmed the successful expression of BoV-ATPase A protein in transfected Sf9 cells (Figure 4A,B, File S1). In the absence of toxin treatment, the mortality rates of cells transfected with the empty vector remained consistently low (<7%). Upon exposure to activated Cry39Ab1 protein, BoV-ATPase A overexpressing cells exhibited a dose-dependent increase in cell death, characterized by swelling, loss of normal shape, and trypan blue uptake. Treatment with 10 μg/mL and 100 μg/mL of activated Cry39Ab1 resulted in mortality rates of 19.15% and 49.41%, respectively, which were significantly higher than those in empty vector-transfected controls (*p* < 0.01) (Figure 4C,D). These results demonstrate that overexpression of BoV-ATPase A markedly enhances the susceptibility of Sf9 cells to activated Cry39Ab1.

### 3.5. RNAi-Mediated Silencing of BoV-ATPase A Reduces the Susceptibility of B. odoriphaga to Cry39Ab1

**Figure 5:**
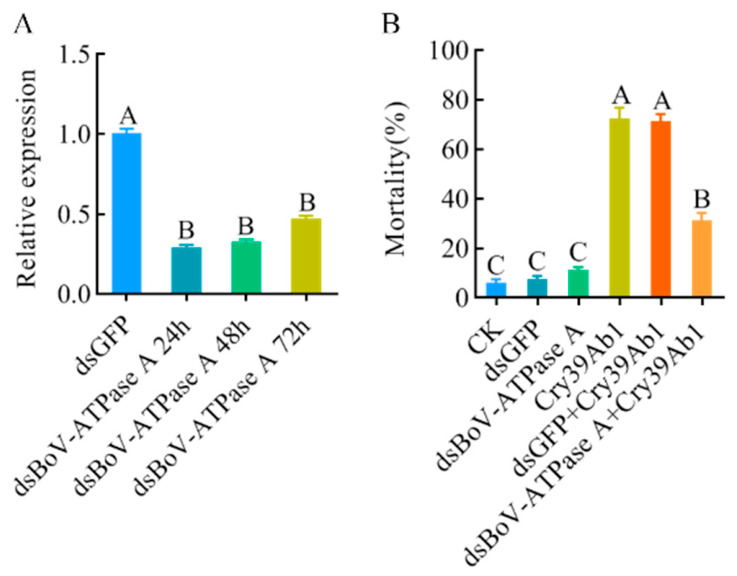
Silencing of BoV-ATPase A reduces susceptibility to Cry39Ab1. (**A**) Knockdown efficiency of *BoV-ATPase A.* (**B**) Final mortality rates (72 h) of larvae subjected to the six indicated treatments: 1. Untreated control; 2. dsGFP injection; 3. dsBoV-ATPase A injection; 4. Cry39Ab1-treated diet; 5. dsGFP injection + Cry39Ab1 diet; 6. dsBoV-ATPase A injection + Cry39Ab1 diet. Each bar represents the mean ± SD of at least three independent replicates (20 larvae per replicate). Bars with different capital (*p* < 0.01) letters differ significantly (means were separated by LSD test, DPSSOFT: DPS7.05).

We employed RNAi to silence *BoV-ATPase A* expression in second-instar larvae. qRT-PCR analysis confirmed a significant reduction (>50%) in *BoV-ATPase A* transcript levels at 24, 48, and 72 h post-feeding, compared to the dsGFP (Figure 5A).

The impact of this silencing on Cry39Ab1 susceptibility was assessed through a bioassay comprising six distinct treatments (Figure 5B). As expected, larvae fed on a normal artificial diet supplemented with dsGFP or dsBoV-ATPase A, exhibited negligible mortality (<10%), suggesting the lack of inherent toxicity from the dsRNA treatments. Larvae exposed to Cry39Ab1 alone suffered high mortality, reaching 72.5% at 72 h post-treatment. Combined treatment with dsGFP and Cry39Ab1 resulted in a similar mortality rate (70.0%), indicating that the RNAi did not affect toxin potency. In contrast, the simultaneous delivery of dsBoV-ATPase A and Cry39Ab1 caused a significant reduction in larval mortality to 31.2% (*p* < 0.01). These data suggest that BoV-ATPase A is a critical component for Cry39Ab1 toxin activity *in vivo*.

## 4. Discussion

V-ATPase is a key proton pump that is widely present and functionally conserved in insects, and its genes have been identified in multiple insect orders such as Coleoptera [29], Lepidoptera [21], Diptera [30] and Orthoptera [31]. The *BoV-ATPase A* was cloned and characterized from *B. odoriphaga* (Figure 1A). Phylogenetic analysis positioned BoV-ATPase A within a clade predominantly containing dipteran orthologs, such as those from *Anopheles bellator* and *Drosophila melanogaster*, consistent with its taxonomic classification (Figure 1B). Quantitative expression profiling across developmental stages revealed the highest transcript abundance in the third-instar larvae, followed by the second instar (Figure 2), and this pattern also was observed in *Plutella xylostella* [32]. We hypothesize that the elevated expression during the second and third instars correlates with heightened physiological demands, including intensive feeding, rapid growth, nutrient assimilation, and osmoregulation. The subsequent decline in expression in later instars and pupae suggests tight developmental regulation of *BoV-ATPase A*, linking its primary function to larval-specific processes of active metabolism and ion homeostasis.

In this study, we provide multiple lines of evidence that BoV-ATPase A is capable of binding to Cry39Ab1 and mediating its insecticidal activity. First, a direct and specific interaction between BoV-ATPase A and Cry39Ab1 was confirmed *in vitro* by GST pull-down assays (Figure 3). Second, heterologous expression of BoV-ATPase A conferred Sf9 cells with specific susceptibility to Cry39Ab1 (Figure 4). Finally, *in vivo* knockdown of *BoV-ATPase A* significantly reduced the larval mortality of *B. odoriphaga* upon Cry39Ab1 exposure, demonstrating that the toxin’s potency dependents on the expression level of this receptor (Figure 5). These findings align with previous reports implicating V-ATPase subunits in the mode of action of Cry toxins in other insects. For instance, RNAi targeting *V-ATPase A* decreased the susceptibility of *C. suppressalis* to Cry1Ca and Cry2Aa [21], while expression of V-ATPase B or E subunits in cell lines enhanced sensitivity to Cry1Ac and Cry2Ab [20,33]. Collectively, our data strongly support the role of BoV-ATPase A as a key mediator of Cry39Ab1 toxicity in *B. odoriphaga*.

The insect midgut V-ATPase is a central driver of luminal alkalization and ion homeostasis. By pumping protons (H^+^) into the gut lumen, it generates a positive transepithelial potential that subsequently energizes the electroneutral K^+^/2H^+^ antiporter, thereby maintaining the characteristic alkaline environment in lepidopteran and dipteran larvae [14,34]. Notably, Cry1 toxins have been shown to cause rapid, dose-dependent depolarization of this potential, directly correlating with their insecticidal potency [23,33,35]. V-ATPase A is a component of the cytosolic V_1_ domain and is not exposed on the cell surface [14]. Therefore, it cannot function as a direct initial binding receptor for extracellular Cry39Ab1. Instead, our data indicate that BoV-ATPase A is an essential intracellular factor for Cry39Ab1 toxicity, but the precise mechanism (direct or indirect) remains to be determined.

One possibility is a direct intracellular interaction. Although speculative, Cry39Ab1 could be internalized via other primary receptors (cadherin or GPI-anchored proteins) and then physically interact with BoV-ATPase A on intracellular vesicles or endosomal membranes, potentially disrupting proton pump activity and ion homeostasis. However, our current data do not provide direct evidence for such an internalization or colocalization event, and this model requires future experimental testing (endocytosis inhibition, toxin trafficking studies). An alternative, and equally plausible, explanation is an indirect effect. Knockdown of *BoV-ATPase A* by RNAi could alter the physiological state of gut epithelial cells—for example, by disrupting luminal pH or intracellular ion balance, thereby indirectly reducing susceptibility to Cry39Ab1. Similarly, heterologous expression of BoV-ATPase A in Sf9 cells might modify cellular homeostasis in a way that facilitates toxin action, without requiring the protein to act as a bona fide receptor. Our current experimental data cannot distinguish between these direct and indirect mechanisms.

Our findings establish that BoV-ATPase A is a key protein required for Cry39Ab1 toxicity in *B. odoriphaga*. This is the first report identifying a functional role for V-ATPase A in the action of a Cry39Ab1 toxin. Future studies should investigate whether endocytosis is required for Cry39Ab1 toxicity, examine the subcellular localization of the toxin and its colocalization with BoV-ATPase A, assess the indirect effects on gut pH and ion homeostasis, use quantitative cytotoxicity assays (LDH or MTT), test an ortholog from a Cry39Ab1-insensitive insect, and perform histological analyses to identify the specific gut cell types targeted by the toxin. These experiments will help distinguish between direct and indirect mechanisms and fully elucidate the role of BoV-ATPase A in Cry39Ab1 action.

## Figures and Tables

**Table 1:** Primers in this study.

Primer Name	Applications	Sequence (5′ → 3′)
V-ATPase A-F	Cloning	ATGTCGTTCCTTTCATACCGTTTAGCC
V-ATPase A-R		CACAAAATTCTTGAGCACATTCTCACAGTAA
M13-F		CAGGGTTTTCCCAGTCACG
M13-R		GAGCGGATAACAATTTCACAC
gst-cry39Ab1-F	Prokaryotic expression	GGTTCCGCGTGGATCCCCGGAATTCATGAATTCATACGAGAATAAAAATGAATATG
gst-cry39Ab1-R		GTCAGTCACGATGCGGCCGCTCGAGTTAATTGGTAAACAGATCGTTCACC
his-cry39Ab1-F		AACTTTAAGAAGGAGATATACCATGGATGAATTCATACGAGAATAAAAATGAATAT
his-cry39Ab1-R		CAGTGGTGGTGGTGGTGGTGCTCGAGATTGGTAAACAGATCGTTCACC
gst-V-ATPase A-F		TCTGGTTCCGCGTGGATCCCCGGAATTCATGTCGTTCCTTTCATACCGTTTAGCC
gst-V-ATPase A-R		AGATCGTCAGTCAGTCACGATGCGGCCGCTCGAGTTACTGTGAGAATGTGCTCAAG
his-V-ATPase A-F		GTTTAACTTTAAGAAGGAGATATACCATGGATGTCGTTCCTTTCATACCGTTTAGCC
his-V-ATPase A-R		GGATCTCAGTGGTGGTGGTGGTGGTGCTCGAGCTGTGAGAATGTGCTCAAGAATT
pGEX5		GGGCTGGCAAGCCACGTTTGGTG
pGEX3		CCGGGAGCTGCATGTGTCAGAGG
T7		TAATACGACTCACTATAGGG
T7-ter		GCTAGTTATTGCTCAGCGG
dsV-ATPase A-F1	RNAi	GCGTAATACGACTCACTATAGGCCGTGTATTGAGCATTGGTG
dsV-ATPase A-R1		GCGTACAGACACACGAGGAA
dsV-ATPase A-F2		CCGTGTATTGAGCATTGGTG
dsV-ATPase A-R2		GCGTAATACGACTCACTATAGGGCGTACAGACACACGAGGAA
dsGFP-F1		GCGTAATACGACTCACTATAGGTGGTCCCAATTCTCGTGGAAC
dsGFP-R1		CTTGAAGTTGACCTTGATGCC
dsGFP-F2		TGGTCCCAATTCTCGTGGAAC
dsGFP-R2		GCGTAATACGACTCACTATAGGCTTGAAGTTGACCTTGATGCC
qV-ATPase A-F	qRT-PCR	TTGTATCCGCTACCGCTTCC
qV-ATPase A-R		TGGTGGACGACGCAACAATA
EF1a-F		TGCAACTGCACTGCGAAAAG
EF1a-R		ACACTTTGCCCTACCGTCTG
RPL18-F		CCAACTGGCAAGGGAACTCT
RPL18-R		AGCTACGTCTGCGACCTCTA

## Data Availability

The original contributions presented in this study are included in the article. Further inquiries can be directed to the corresponding authors.

## Supplementary Materials

The following supporting information can be downloaded at: https://www.mdpi.com/article/10.3390/insects17060563/s1, File S1: Original Images for Blots.
