# Supplementary figures and images for: V-ATPase A Is a Key Protein Involved in the Toxicity of Bacillus thuringiensis Cry39Ab1 in Bradysia odoriphaga (Diptera: Sciaridae)
Source: Insects. 2026 May 29;17(6):563. doi: 10.3390/insects17060563 (PMC13299436; doi:10.3390/insects17060563)

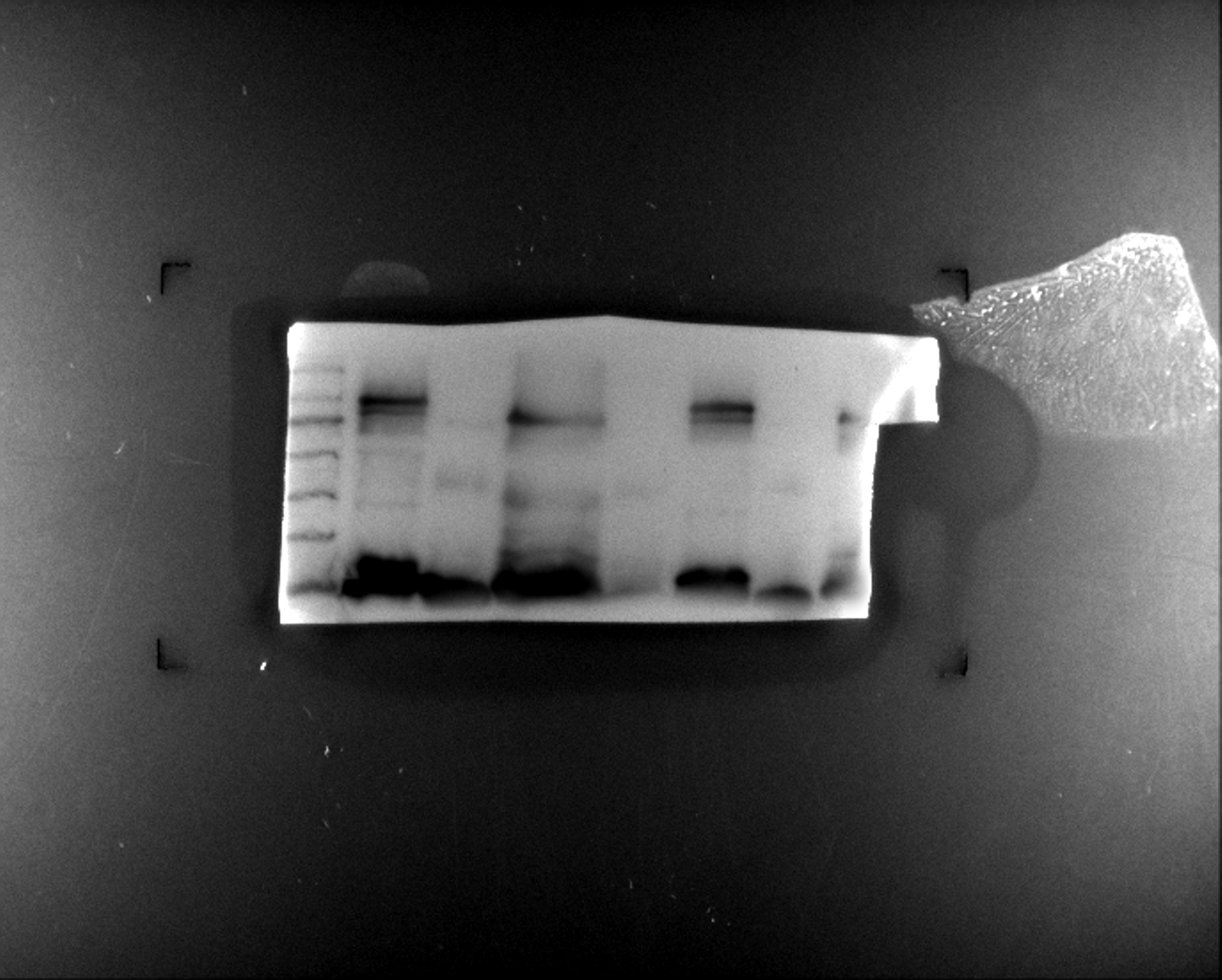

Supplement: Supplementary file 1 [file insects-17-00563-s001.zip › File S1/Figure3E/Input-GST-Cry39Ab1+GST-V-ATPase A.tif]

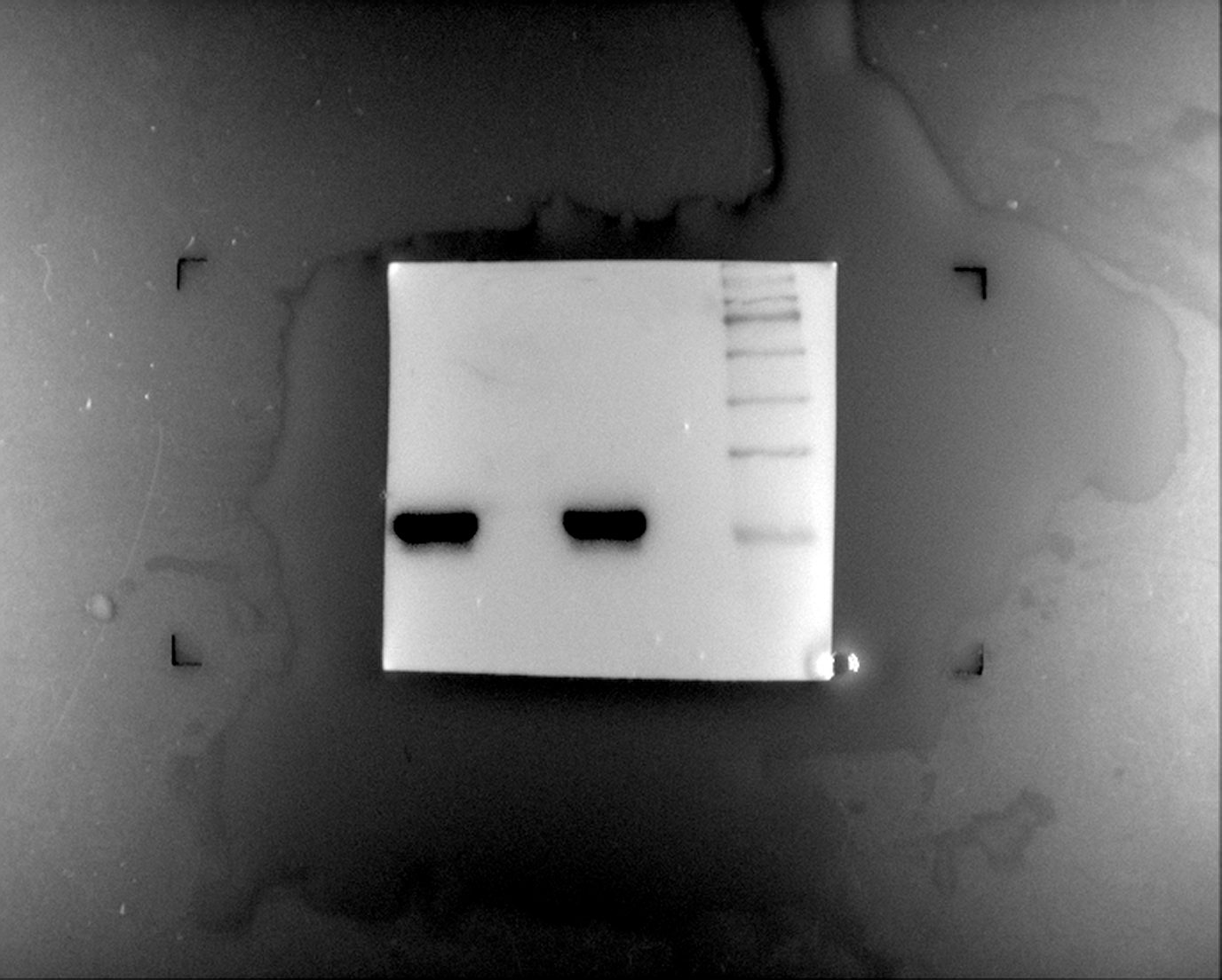

Supplement: Supplementary file 1 [file insects-17-00563-s001.zip › File S1/Figure3E/input-GST.tif]

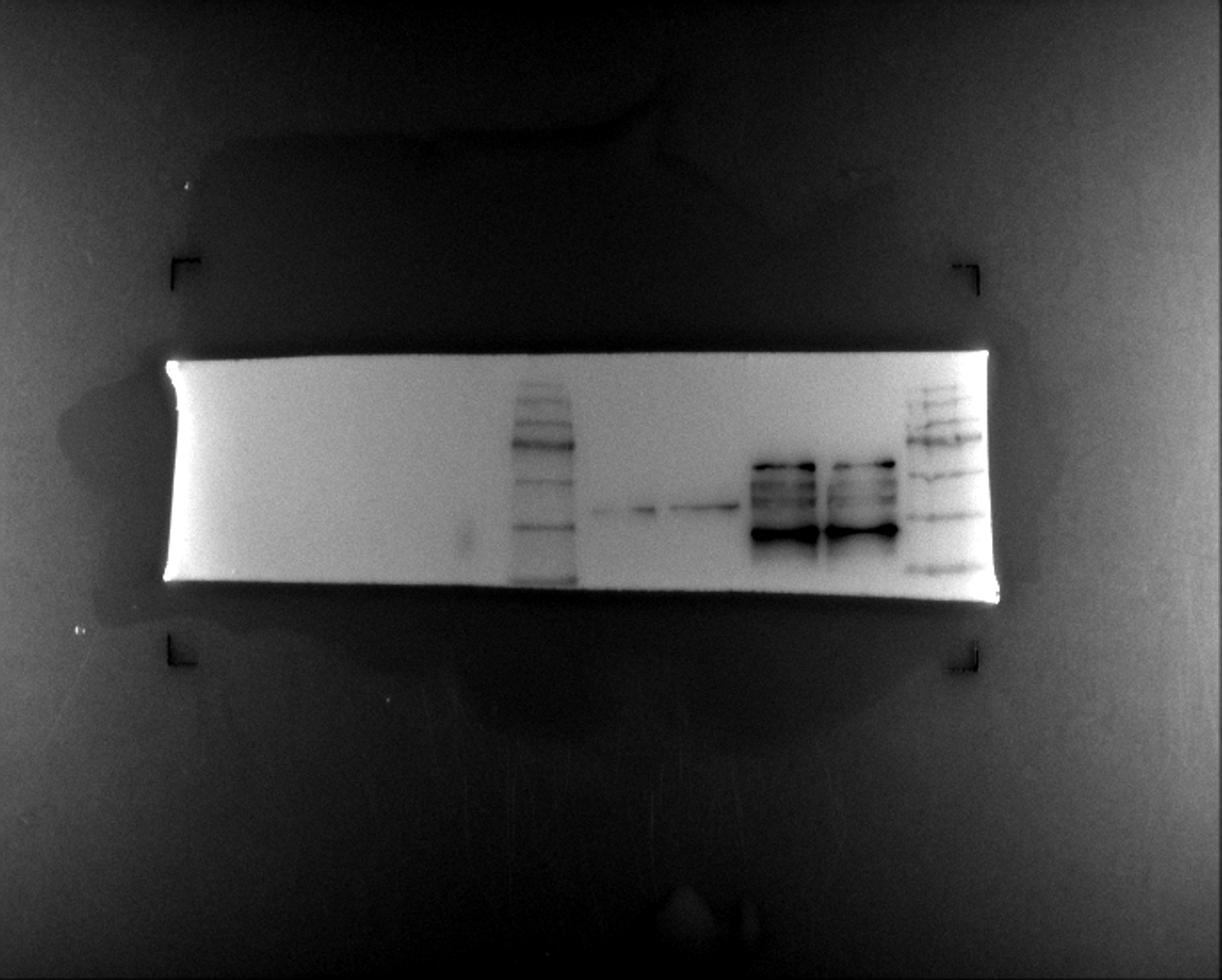

Supplement: Supplementary file 1 [file insects-17-00563-s001.zip › File S1/Figure3E/Input-HisV-ATPase A+His-Cry39Ab1.tif]

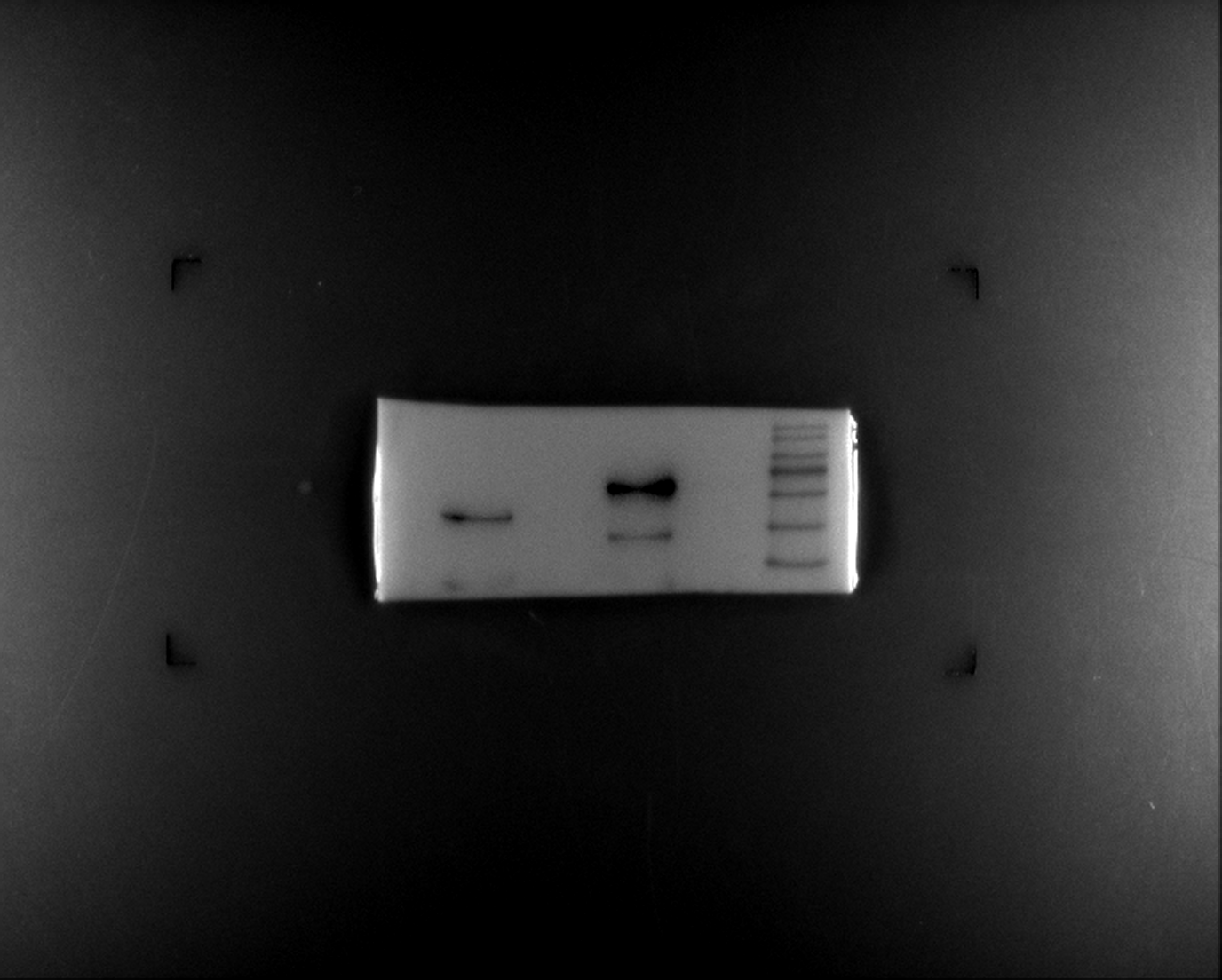

Supplement: Supplementary file 1 [file insects-17-00563-s001.zip › File S1/Figure3E/pull-down.tif]

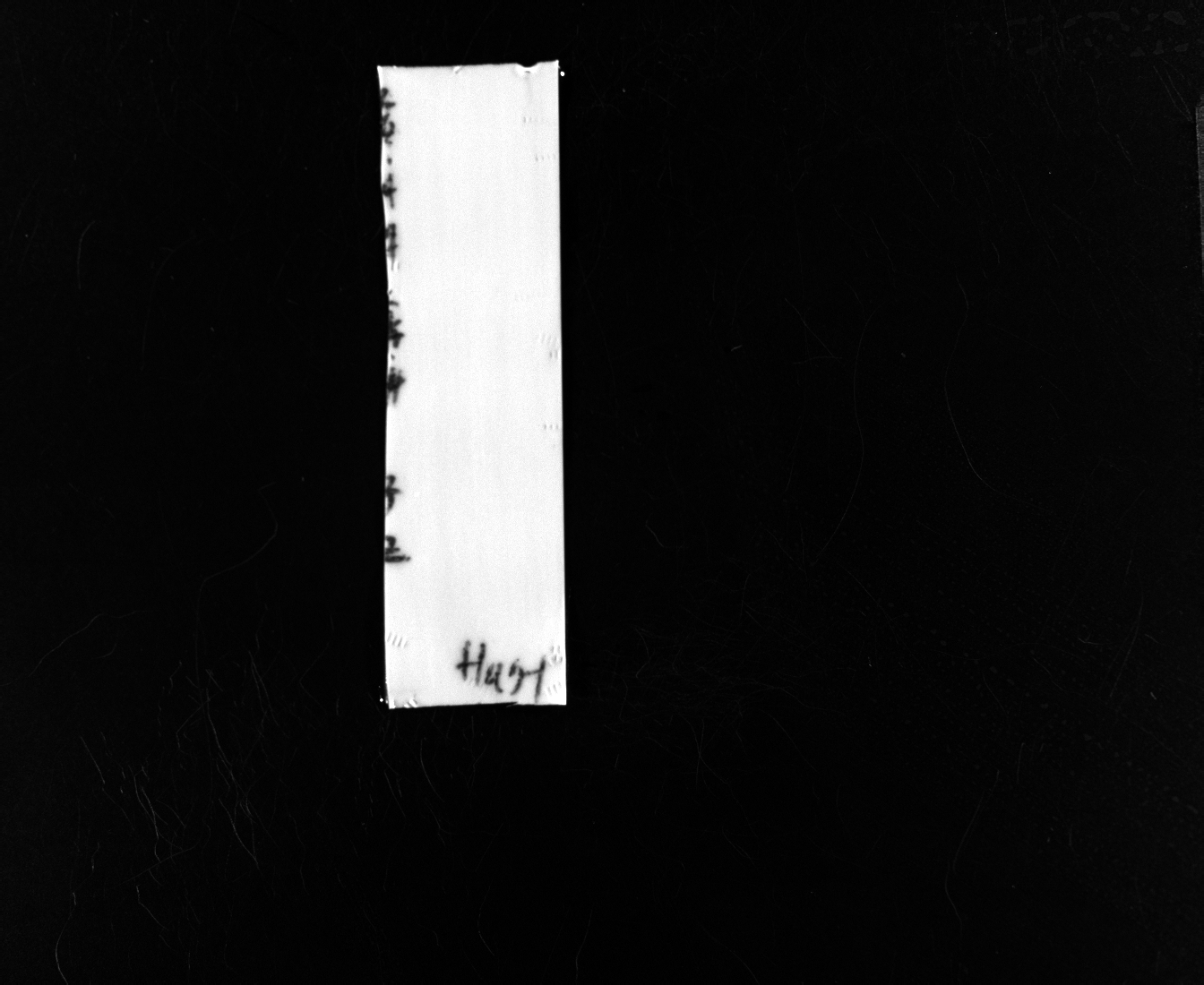

Supplement: Supplementary file 1 [file insects-17-00563-s001.zip › File S1/Figure4B/004[FLAG-1]-20251219-105047-brightfield.tif]

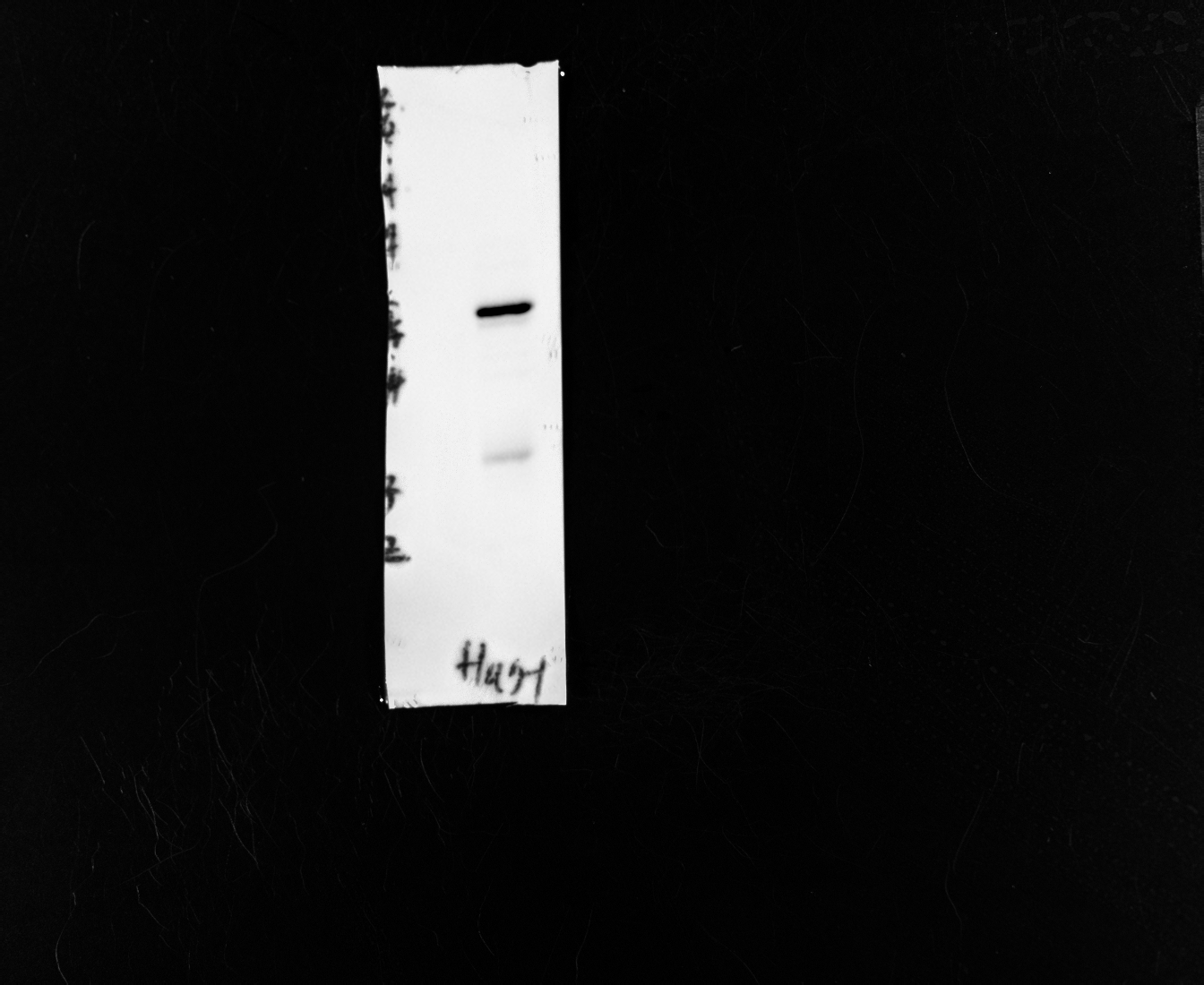

Supplement: Supplementary file 1 [file insects-17-00563-s001.zip › File S1/Figure4B/004[FLAG-1]-20251219-105047-luminescence-overlay.tif]

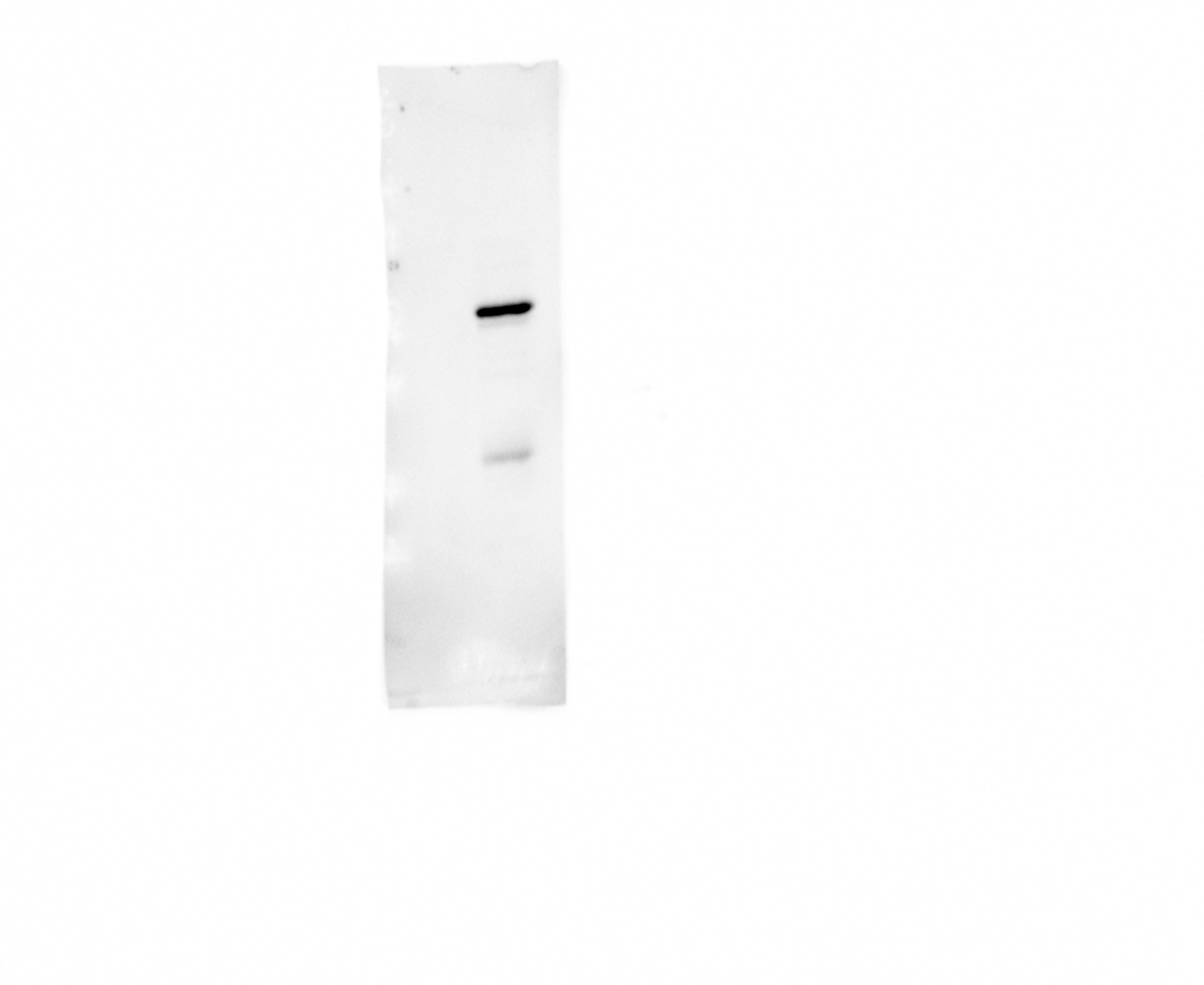

Supplement: Supplementary file 1 [file insects-17-00563-s001.zip › File S1/Figure4B/004[FLAG-1]-20251219-105047-luminescence.tif]

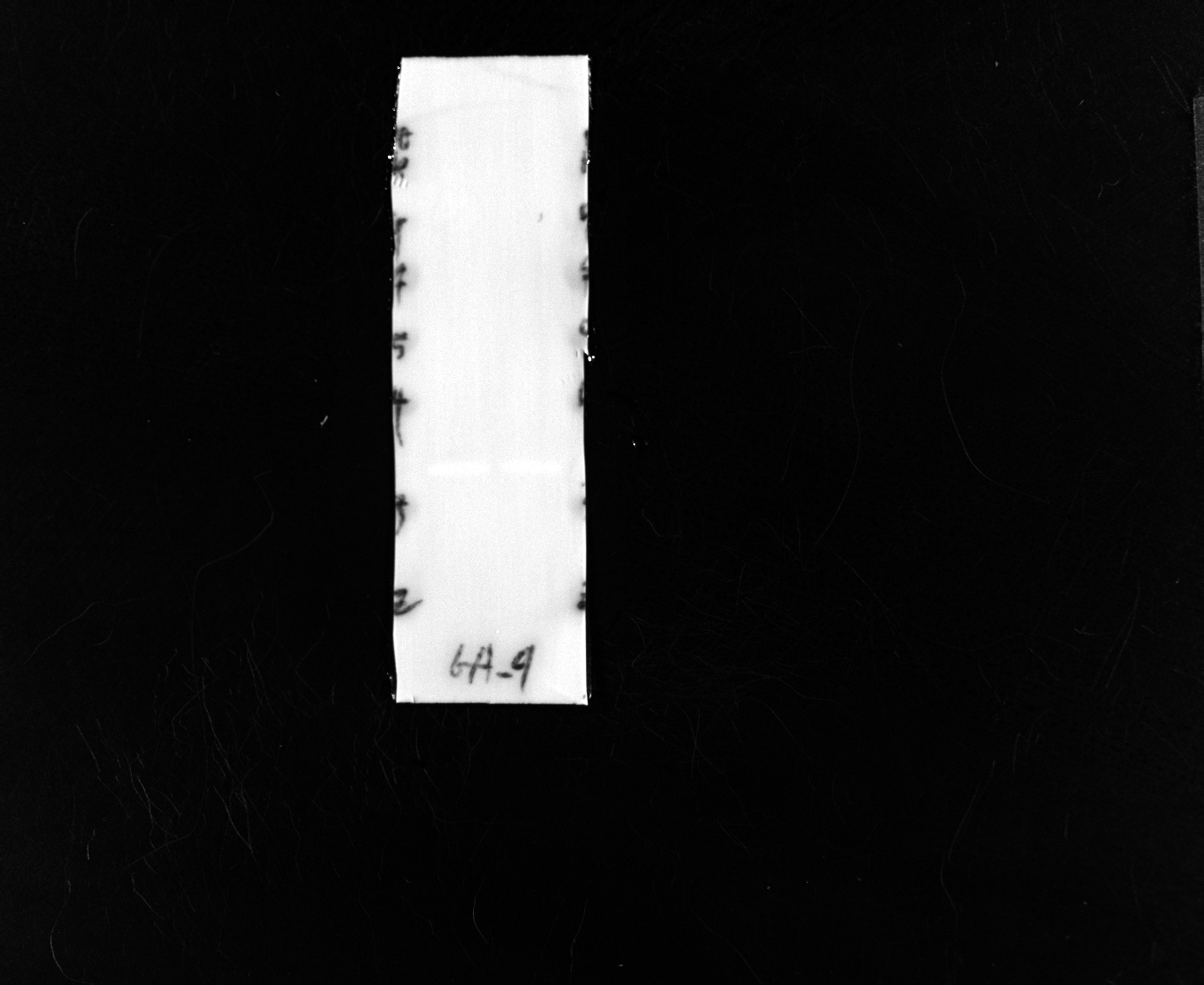

Supplement: Supplementary file 1 [file insects-17-00563-s001.zip › File S1/Figure4B/011[GAPDH-9]-20251219-104503-brightfield.tif]

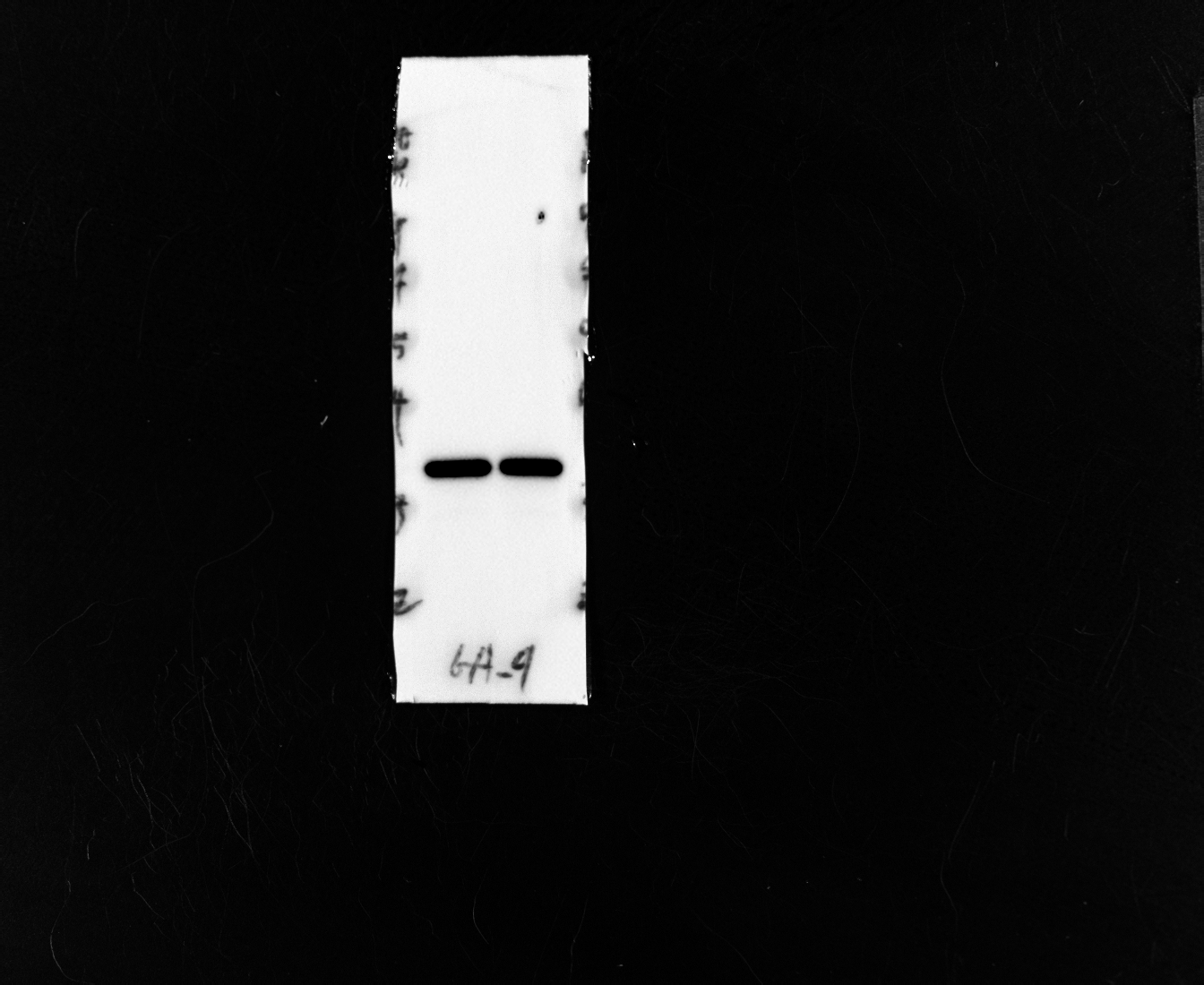

Supplement: Supplementary file 1 [file insects-17-00563-s001.zip › File S1/Figure4B/011[GAPDH-9]-20251219-104503-luminescence-overlay.tif]

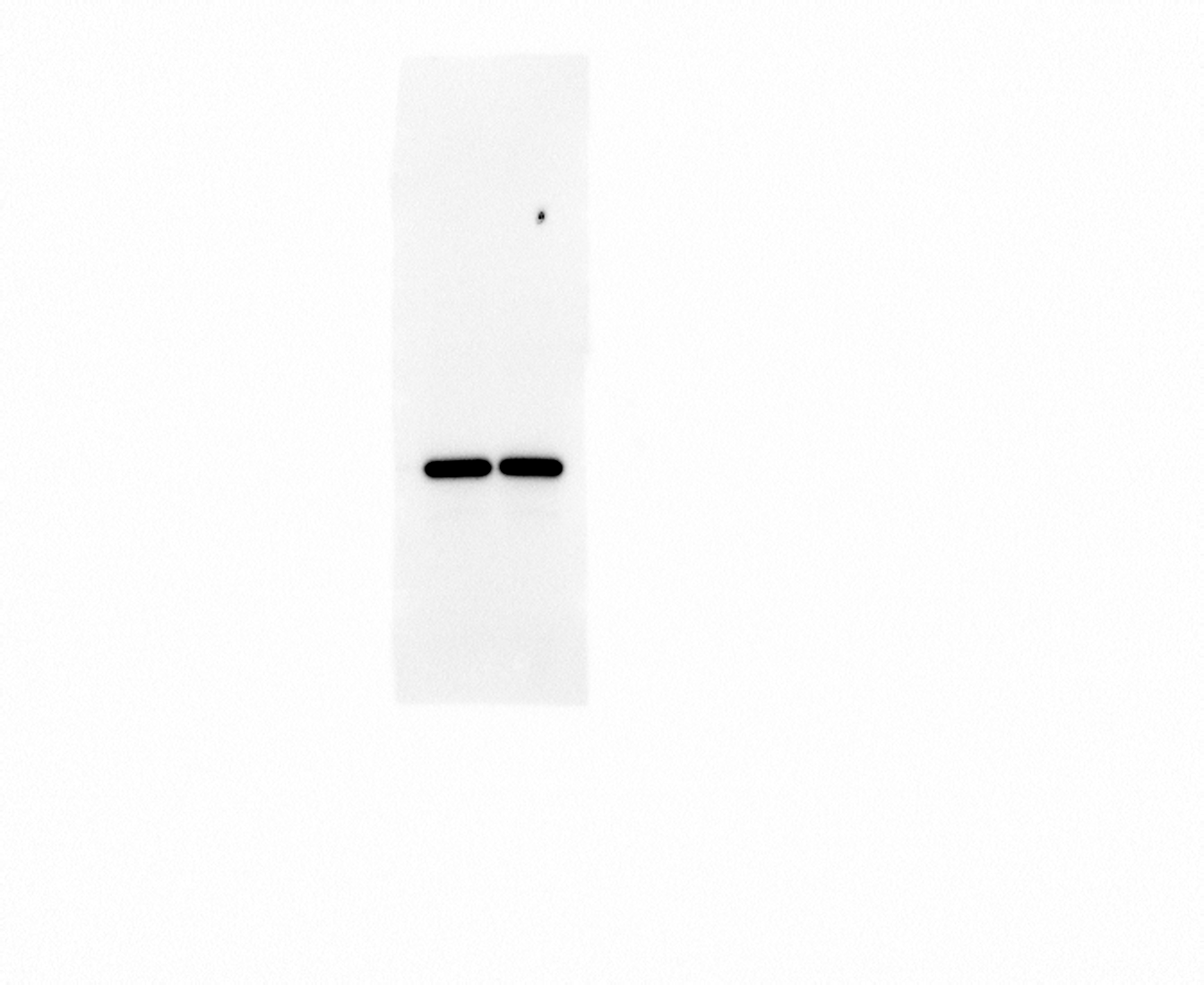

Supplement: Supplementary file 1 [file insects-17-00563-s001.zip › File S1/Figure4B/011[GAPDH-9]-20251219-104503-luminescence.tif]
